# A retrospective analysis of clinical outcomes between hospitalized patients with COVID‐19 who received famotidine or pantoprazole

**DOI:** 10.1002/jgh3.12905

**Published:** 2023-07-10

**Authors:** Justin J. Wagner, Nikolas St. Cyr, Aaron Douen, Joshua Fogel, John Trillo

**Affiliations:** ^1^ Department of Medicine South Brooklyn Health Brooklyn New York USA; ^2^ Department of Business Management Brooklyn New York USA

**Keywords:** COVID‐19, famotidine, pantoprazole, mortality, artificial respiration, acute kidney injury, gastrointestinal hemorrhage

## Abstract

**Background and Aim:**

There is limited research on the use of histamine‐H2 receptor antagonists and proton pump inhibitors for treating COVID‐19. We compare clinical outcomes between patients hospitalized with COVID‐19 receiving famotidine or pantoprazole.

**Methods:**

This retrospective study included 2184 patients (famotidine: *n* = 638, pantoprazole: *n* = 727, nonuse: *n* = 819) aged 18 years or older treated for COVID‐19 from March 2020 to March 2021. Patients who received both famotidine and pantoprazole treatments were excluded. Multivariate logistic regression was used for the primary outcome, namely all‐cause mortality, and the secondary outcomes, namely mechanical ventilation, vasopressor use, acute kidney injury, and gastrointestinal bleeding. The main predictor variable was the use of famotidine or pantoprazole. Covariates were demographics, chronic diseases, and symptoms.

**Results:**

As compared to nonuse, famotidine (OR: 0.30, 95% CI: 0.20–0.44, *P* < 0.001) and pantoprazole (OR: 0.47, 95% CI: 0.33–0.66, *P* < 0.001) were significantly associated with lower odds for all‐cause mortality. Comparison of famotidine and pantoprazole showed that the former had lower odds for all‐cause mortality (OR: 0.65, 95% CI:0.45–0.95, *P* < 0.05), mechanical ventilation (OR: 0.38, 95% CI: 0.25–0.58, *P* < 0.001), vasopressor use (OR: 0.33, 95% CI: 0.22–0.48, *P* < 0.001), acute kidney injury (OR: 0.40, 95% CI: 0.30–0.54, *P* < 0.001), and gastrointestinal bleeding (OR: 0.15, 95% CI: 0.08, 0.29, *P* < 0.001).

**Conclusions:**

Famotidine is associated with lower odds for all‐cause mortality, mechanical ventilation, vasopressor use, acute kidney injury, and gastrointestinal bleeding as compared to pantoprazole in patients hospitalized with COVID‐19. We recommend that clinicians consider the use of famotidine over pantoprazole for hospitalized COVID‐19 patients. Future research with a clinical trial would be beneficial to further support such use of famotidine.

## Graphical abstract

We compare clinical outcomes between patients hospitalized with COVID‐19 receiving famotidine or pantoprazole. Famotidine is associated with lower odds for all‐cause mortality, mechanical ventilation, vasopressor use, acute kidney injury, and gastrointestinal bleeding as compared to pantoprazole in patients hospitalized with COVID‐19. We recommend that clinicians consider the use of famotidine over pantoprazole for hospitalized COVID‐19 patients.

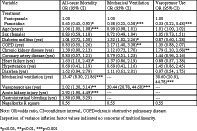

## Introduction

As of January 2023, severe acute respiratory syndrome coronavirus 2 (SARS‐CoV‐2), which causes coronavirus disease 2019 (COVID‐19), has resulted in over 661 million cases and more than 6.7 million deaths worldwide.^1^ The spectrum of illness among patients with COVID‐19 ranges from asymptomatic to mild to severe, with varied clinical features involving the respiratory, cardiovascular, gastrointestinal, and renal systems and resulting in mortality, mechanical ventilation, vasopressor use, acute kidney injury, and gastrointestinal bleeding.^2^ The histamine‐H2 receptor antagonist famotidine has been of interest for treating patients with COVID‐19.

It is hypothesized that famotidine potentially inhibits 3‐chymotrypsin‐like protease (3CLpro), which transforms proteins that are fundamental for viral replication.^3^ Some studies have used computer and bioinformatic modeling to hypothesize that famotidine, as a histamine‐H2 receptor antagonist, could inhibit 3CLpro.^4^ This immunomodulatory activity of famotidine may be inhibiting the pathological histamine signaling in immunoregulation and inflammation, which may be one of the primary factors causing severe SARS‐CoV‐2 infection.^4^ However, others have opposed this hypothesis and postulated that famotidine does not act as an antiviral inhibitor of the proteases of SARS‐CoV‐2.^5^ Consequently, the efficacy of famotidine use for improving clinical outcomes of patients with COVID‐19 remains controversial.

The initial impetus for the study of famotidine in COVID‐19 patients came from an anecdotal report from Wuhan, China, which correlated famotidine use with COVID‐19 survival.^6^ Additional studies found an association between famotidine use and the reduction in mortality or mechanical ventilation in patients with COVID‐19.^7^, ^8^ In contrast, other studies observed no association between famotidine use and the risk of mortality or mechanical ventilation in patients with COVID‐19.^9^, ^10^, ^11^ Furthermore, a systemic review and meta‐analysis of these five studies determined that there were no significant protective effects of famotidine in patients with COVID‐19, but argued that more original studies were needed to evaluate the association between famotidine use and reduced severity of infection, mortality, or mechanical ventilation.^12^

Proton pump inhibitors (PPIs), such as pantoprazole, have an important role in the therapeutic approach to acid‐related disorders and are very commonly prescribed. PPIs can inhibit pro‐inflammatory cytokines, similar to histamine‐H2 receptor antagonists such as famotidine, which can potentially decrease the cytokine storm associated with severe COVID‐19.^13^ However, in contrast to histamine‐H2 receptor antagonists, bacterial overgrowth of the upper gastrointestinal tract can occur when gastric acid production is reduced with the use of PPIs, which may trigger the development of pneumonia.^14^ Consequently, multiple studies have demonstrated a significantly increased odds for severe COVID‐19 infection with the use of PPIs in comparison to its nonuse.^15^, ^16^, ^17^ Furthermore, a systemic review and meta‐analysis argued that although the increased risk for a severe COVID‐19 infection was small with the use of PPIs, the decision to discontinue PPIs should be made on an individual basis, and more original studies would be needed to evaluate the association between PPI use and increased severity of infection, death, and intubation.^18^

We are not aware of any head‐to‐head study directly comparing famotidine to pantoprazole for treating COVID‐19. We retrospectively compare the impact of famotidine use, pantoprazole use, and use of neither drug on the primary outcome of all‐cause mortality and the secondary outcomes of mechanical ventilation, vasopressor use, acute kidney injury, and gastrointestinal bleeding for patients hospitalized with COVID‐19.

## Materials and methods

This retrospective study included 2184 patients aged 18 years or older who were admitted to a public community hospital located in Brooklyn, NY, between March 1, 2020 and March 1, 2021, and who tested positive for SARS‐CoV‐2 by polymerase chain reaction within 72 h of presentation. Institutional review board approval was granted, and informed consent was waived because of the retrospective nature of this study.

The three patient groups were categorized by famotidine use, pantoprazole use, or nonuse. Famotidine and pantoprazole use were designated if administered within 24 h of hospitalization whether through intravenous or oral administration. Use included any dose, duration, or frequency. Nonuse was if neither famotidine nor pantoprazole was used. Patients who received both famotidine and pantoprazole or who received either medication more than 24 h after hospitalization were excluded.

Demographic characteristics included age (years) and sex (male/female). Comorbid conditions included diabetes mellitus, chronic obstructive pulmonary disease (COPD), chronic kidney disease, coronary artery disease, congestive heart failure, and hypertension, all measured as no/yes. We also recorded presence of diarrhea (no/yes). The primary outcome variable was all‐cause mortality. Secondary outcome variables were mechanical ventilation, vasopressor use, acute kidney injury, and gastrointestinal bleeding.

Mean and standard deviation were used to describe the continuous variable (age). Frequency and percentage were used to describe the categorical variables. Analysis of variance compared the continuous variable (age) among the groups. The Pearson chi‐square statistic compared the categorical variables among the groups. Multivariate logistic regression was conducted for the primary outcome of all‐cause mortality and the secondary outcomes of mechanical ventilation, vasopressor use, acute kidney injury, and gastrointestinal bleeding. SPSS Statistics v. 26 (IBM Corporation, Armonk, NY) was used for analyses. All *P*‐values were two‐tailed. Alpha level for statistical significance was set at *P* < 0.05.

## Results

The study groups consisted of users of none (37.5%, *n* = 819), famotidine (29.2%, *n* = 638), and pantoprazole (33.3%, *n* = 727). Table 1 compares baseline demographic characteristics, medical symptoms and history, and the outcomes among the study groups. For demographics, both mean age (*P* < 0.001) and sex (*P* = 0.02) significantly differed among the groups, with the pantoprazole group having the greatest mean age and the lowest percentage of females. For medical symptoms and history, all variables had significantly different percentages among the groups (all *P* < 0.001) except for diarrhea. The overall pattern for these percentage differences was that pantoprazole had the greatest percentage, and no treatment had the lowest percentage except for chronic kidney disease where famotidine had the lowest percentage and pantoprazole and no treatment had very similar percentages. For outcomes, all had significantly different percentages among the groups (all *P* < 0.001). The overall pattern for these differences was that pantoprazole had the highest percentage and famotidine had the lowest percentage.

**Table 1:** Baseline demographic characteristics, medical symptoms and history, and outcomes in the study groups

Variable	None M (SD) or frequency (%) (*n* = 819)	Famotidine M (SD) or frequency (%) (*n* = 638)	Pantoprazole M (SD) or frequency (%) (*n* = 727)	*P*‐value
Demographics				
Age [years] (mean)	57.7 (21.01)	64.9 (18.80)	67.3 (17.20)	<0.001
Sex (female)	403 (49.2)	293 (46.2)	305 (42.0)	0.02
Medical symptoms and history				
Diabetes mellitus (yes)	378 (46.2)	307 (48.1)	440 (60.5)	<0.001
COPD (yes)	77 (9.4)	89 (13.9)	156 (21.5)	<0.001
Chronic kidney disease (yes)	257 (31.4)	117 (18.3)	230 (31.6)	<0.001
Coronary artery disease (yes)	136 (16.6)	141 (22.1)	250 (34.4)	<0.001
Heart failure (yes)	84 (10.3)	117 (18.3)	168 (23.1)	<0.001
Hypertension (yes)	460 (56.2)	464 (72.7)	574 (79.0)	<0.001
Diarrhea (yes)	66 (8.1)	71 (11.1)	70 (9.6)	0.14
Outcomes				
All‐cause mortality (yes)	182 (22.2)	82 (12.9)	255 (35.1)	<0.001
Mechanical ventilation (yes)	75 (9.2)	48 (7.5)	234 (32.2)	<0.001
Vasopressor use (yes)	98 (12.0)	55 (8.6)	261 (35.9)	<0.001
Acute kidney injury (yes)	255 (31.1)	196 (30.7)	399 (54.9)	<0.001
Gastrointestinal bleeding (yes)	24 (2.9)	12 (1.9)	84 (11.6)	<0.001

COPD, chronic obstructive pulmonary disease; M, mean; SD, standard deviation.

Table 2 shows the results of multivariate logistic regression analyses for all‐cause mortality, mechanical ventilation, and vasopressor use among the famotidine, pantoprazole, and nonuse groups. Both famotidine (OR: 0.30, 95% CI: 0.20–0.44, *P* < 0.001) and pantoprazole (OR: 0.47, 95% CI: 0.33–0.66, *P* < 0.001) were significantly associated with lower odds for all‐cause mortality. Pantoprazole was significantly associated with higher odds for mechanical ventilation (OR: 2.53, 95% CI: 1.77–3.64, *P* < 0.001) while famotidine was not. Also, pantoprazole was significantly associated with higher odds for vasopressor use (OR: 1.94, 95% CI: 1.39–2.71, *P* < 0.001), while famotidine was significantly associated with lower odds (OR: 0.62, 95% CI: 0.41–0.95, *P* < 0.05).

**Table 2:** Multivariate logistic regression for the primary outcome of all‐cause mortality and the secondary outcomes of mechanical ventilation and vasopressor use among famotidine, pantoprazole, and nonuse groups

Variable	All‐cause mortality OR (95% CI)	Mechanical ventilation OR (95% CI)	Vasopressor use OR (95% CI)
Treatment			
None	1.00	1.00	1.00
Famotidine	0.30 (0.20–0.44)***	0.93 (0.59–1.47)	0.62 (0.41–0.95)*
Pantoprazole	0.47 (0.33–0.66)***	2.53 (1.77, 3.64)***	1.94 (1.39, 2.71)***
Age (years)	1.08 (1.07, 1.09)***	1.01 (1.00–1.02)	1.01 (1.004–1.02)**
Sex (female)	0.91 (0.68–1.21)	0.57 (0.42–0.78)***	1.14 (0.85–1.53)
Diabetes mellitus (yes)	0.92 (0.69–1.24)	1.32 (0.95–1.84)	1.10 (0.81–1.49)
COPD (yes)	0.94 (0.66–1.33)	1.88 (1.29–2.74)**	1.47 (1.02–2.11)*
Chronic kidney disease (yes)	1.33 (0.93–1.88)	1.10 (0.78–1.55)	1.43 (1.04–1.96)*
Coronary artery disease (yes)	0.86 (0.62–1.19)	0.65 (0.45–0.95)*	1.49 (1.06–2.10)*
Heart failure (yes)	1.45 (1.03–2.04)*	1.30 (0.87–1.95)	0.91 (0.62–1.33)
Hypertension (yes)	0.65 (0.43–0.97)*	0.85 (0.56–1.28)	1.07 (0.72–1.59)
Diarrhea (yes)	1.37 (0.86–2.17)	1.42 (0.87–2.31)	0.95 (0.59–1.54)
Mechanical ventilation (yes)	14.45 (9.83–21.91)***	—	26.15 (19.15–35.72)***
Vasopressor use (yes)	2.40 (1.66–3.47)	26.18 (19.16–35.79)***	—
Acute kidney injury (yes)	3.42 (2.43–4.82)***	—	—
Gastrointestinal bleeding (yes)	1.47 (0.85–2.56)	—	—
Nagelkerke *R* ^2^	0.56	0.51	0.49

* *P* <0.05;

** *P* <0.01;

*** *P* <0.001.

Inspection of variance inflation factor values indicates no concerns of multicollinearity.

CI, confidence interval; COPD, chronic obstructive pulmonary disease; OR, odds ratio.

Table 3 shows results of multivariate logistic regression analyses for acute kidney injury and gastrointestinal bleeding among the famotidine, pantoprazole, and nonuse groups. Pantoprazole was significantly associated with higher odds for acute kidney injury (OR: 2.02–95% CI: 1.53–2.67, *P* < 0.001) while famotidine was not. Pantoprazole was significantly associated with higher odds for gastrointestinal bleeding (OR: 3.83, 95% CI: 2.32–6.31, *P* < 0.001) while famotidine was not.

**Table 3:** Multivariate logistic regression for the secondary outcomes of acute kidney injury and gastrointestinal bleeding among famotidine, pantoprazole, and nonuse groups

Variable	Acute kidney injury OR (95% CI)	Gastrointestinal bleeding OR (95% CI)
Treatment		
None	1.00	1.00
Famotidine	0.81 (0.60–1.10)	0.59 (0.29–1.20)
Pantoprazole	2.02 (1.53–2.67)***	3.83 (2.32–6.31)***
Age (years)	1.06 (1.05–1.07)***	1.02 (1.01–1.03)**
Sex (female)	0.38 (0.30–0.49)***	1.02 (0.69–1.52)
Diabetes mellitus (yes)	1.39 (1.10–1.76)**	0.78 (0.51–1.20)
COPD (yes)	1.17 (0.85–1.61)	1.29 (0.80–2.09)
Chronic kidney disease (yes)	21.44 (15.21–30.23)***	1.94 (1.26–2.96)**
Coronary artery disease (yes)	1.11 (0.83–1.48)	1.78 (1.14–2.79)*
Heart failure (yes)	1.29 (0.93–1.79)	1.02 (0.62–1.67)
Hypertension (yes)	1.48 (1.09–2.00)*	0.69 (0.40–1.21)
Diarrhea (yes)	1.36 (0.91–2.02)	5.66 (3.59–8.94)***
Nagelkerke *R* ^2^	0.53	0.20

* *P* <0.05;

** *P* <0.01;

*** *P* <0.001.

Inspection of variance inflation factor values indicates no concerns of multicollinearity.

CI, confidence interval; COPD, chronic obstructive pulmonary disease; OR, odds ratio.

Multivariate logistic regression analyses were also performed for the above outcomes directly between famotidine and pantoprazole. Famotidine had significantly lower odds than pantoprazole for all the outcomes, namely all‐cause mortality (OR: 0.65, 95% CI: 0.45–0.95, *P* < 0.05), mechanical ventilation (OR: 0.38, 95% CI: 0.25–0.58, *P* < 0.001), vasopressor use (OR: 0.33, 95% CI: 0.22–0.48, *P* < 0.001) (Table 4), acute kidney injury (OR: 0.40, 95% CI: 0.30–0.54, *P* < 0.001), and gastrointestinal bleeding (OR: 0.15, 95% CI: 0.08–0.29, *P* < 0.001) (Table 5).

**Table 4:** Multivariate logistic regression for the primary outcome of all‐cause mortality and the secondary outcomes of mechanical ventilation and vasopressor use between famotidine and pantoprazole

Variable	All‐cause mortality OR (95% CI)	Mechanical ventilation OR (95% CI)	Vasopressor use OR (95% CI)
Treatment			
Pantoprazole	1.00	1.00	1.00
Famotidine	0.65 (0.45–0.95)*	0.38 (0.25–0.58)***	0.33 (0.22–0.48)***
Age (years)	1.06 (1.05–1.08)***	0.99 (0.98–1.01)	1.01 (1.00–1.02)
Sex (female)	0.83 (0.59–1.18)	0.72 (0.49–1.04)	1.05 (0.73–1.51)
Diabetes mellitus (yes)	1.04 (0.72–1.50)	1.52 (1.02–2.26)*	0.87 (0.60–1.28)
COPD (yes)	0.83 (0.55–1.26)	2.17 (1.40–3.38)**	1.35 (0.88–2.07)
Chronic kidney disease (yes)	1.39 (0.90–2.13)	1.12 (0.73–1.70)	1.79 (1.20–2.68)**
Coronary artery disease (yes)	0.83 (0.57–1.21)	0.79 (0.51–1.23)	1.44 (0.96–2.16)
Heart failure (yes)	1.63 (1.10–2.43)*	1.37 (0.86–2.19)	0.88 (0.57–1.38)
Hypertension (yes)	0.69 (0.41–1.19)	0.69 (0.41–1.16)	1.45 (0.86–2.45)
Diarrhea (yes)	1.62 (0.94–2.79)	1.11 (0.61–2.01)	0.97 (0.54–1.75)
Mechanical ventilation (yes)	13.47 (8.30–21.86)***	—	30.60 (20.91–44.78)***
Vasopressor use (yes)	2.02 (1.30–3.14)**	30.44 (20.78–44.60)***	—
Acute kidney injury (yes)	2.92 (1.90–4.49)***	—	—
Gastrointestinal bleeding (yes)	1.80 (0.98–3.29)	—	—
Nagelkerke *R* ^2^	0.56	0.55	0.55

* *P* <0.05;

** *P* <0.01;

*** *P* <0.001.

Inspection of variance inflation factor values indicates no concerns of multicollinearity.

CI, confidence interval; COPD, chronic obstructive pulmonary disease; OR, odds ratio.

**Table 5:** Multivariate logistic regression for the secondary outcomes of acute kidney injury and gastrointestinal bleeding between famotidine and pantoprazole

Variable	Acute kidney injury OR (95% CI)	Gastrointestinal bleeding OR (95% CI)
Treatment		
Pantoprazole	1.00	1.00
Famotidine	0.40 (0.30–0.54)***	0.15 (0.08–0.29)***
Age (years)	1.05 (1.04–1.06)***	1.01 (1.00–1.03)
Sex (female)	0.51 (0.38–0.69)***	1.39 (0.89–2.18)
Diabetes mellitus (yes)	1.27 (0.94–1.71)	0.62 (0.38–1.01)
COPD (yes)	1.22 (0.84–1.76)	1.36 (0.81–2.28)
Chronic kidney disease (yes)	45.26 (26.91–76.14)***	2.51 (1.54–4.09)***
Coronary artery disease (yes)	1.17 (0.82–1.65)	1.77 (1.07–2.92)*
Heart failure (yes)	1.19 (0.80–1.75)	1.01 (0.59–1.73)
Hypertension (yes)	1.37 (0.92–2.05)	0.83 (0.42–1.64)
Diarrhea (yes)	1.22 (0.75–1.98)	4.63 (2.68–7.80)***
Nagelkerke *R* ^2^	0.55	0.20

* *P* < 0.05;

*** *P* < 0.001.

Inspection of variance inflation factor values indicates no concerns of multicollinearity.

CI, confidence interval; COPD, chronic obstructive pulmonary disease; OR, odds ratio.

## Discussion

In patients hospitalized with COVID‐19, when comparing both famotidine and pantoprazole to nonuse, we found beneficial associations for both famotidine and pantoprazole of significantly lower odds than nonuse for all‐cause mortality. However, pantoprazole had negative associations of significantly increased odds than nonuse for all the other outcomes, namely mechanical ventilation, vasopressor use, acute kidney injury, and gastrointestinal bleeding. Famotidine had an additional beneficial association of significantly lower odds than nonuse for vasopressor use. When comparing famotidine and pantoprazole, the former was more beneficial than the latter, as famotidine had significantly lower odds than pantoprazole for all the outcomes, namely all‐cause mortality, mechanical ventilation, vasopressor use, acute kidney injury, and gastrointestinal bleeding.

We found that both famotidine and pantoprazole were significantly associated with lower odds for all‐cause mortality among COVID‐19 patients, while famotidine had lower odds for all‐cause mortality than pantoprazole. There are mixed findings for famotidine use for COVID‐19 patients, with some reporting an association for reduction in mortality while others reporting no association for mortality.^7^, ^8^, ^9^, ^10^, ^11^ Furthermore, a double‐blinded phase II clinical trial found that famotidine led to earlier symptom resolution in outpatients with COVID‐19 infection.^19^ Our findings are similar to these results reporting a reduction in symptoms and mortality. Various mechanisms could explain the potential clinical benefit of famotidine for COVID‐19 patients. First, famotidine can potentially activate G‐protein‐coupled receptors (GPCRs), which can activate immune cell mobilization and prevent pathological vascular inflammation.^20^ We suggest that owing to the impact of famotidine on possibly preventing vascular inflammation, this results in reduced all‐cause mortality. Second, a computer model has suggested that famotidine is a top‐ranked match for drugs predicted to bind 3‐chymotrypsin‐like protease (3CLpro), which plays a vital role in viral replication.^3^ We suggest that as famotidine has a strong likelihood for binding to a site that promotes viral replication, this can block the adverse impact of COVID‐19 and thus result in reduced all‐cause mortality.

Pantoprazole, which directly blocks the proton pump, is a more potent inhibitor of acid secretion than histamine‐H2 receptor antagonists such as famotidine.^21^ Several meta‐analyses have shown that intravenous administration of PPIs is effective in decreasing the incidence and recurrence of gastrointestinal bleeding due to ulcers in hospitalized patients when compared with placebo or histamine‐H2 receptor antagonists.^22^ We suggest that patients at risk for gastrointestinal bleeding treated with the PPI pantoprazole had this beneficial association of pantoprazole with lower odds for all‐cause mortality as compared to no treatment.

We found that for mechanical ventilation, pantoprazole had higher odds compared to nonuse, while famotidine had lower odds compared to pantoprazole. We also found lower odds for vasopressor use for famotidine as compared to pantoprazole. A meta‐analysis found increased risk with PPI use such as pantoprazole for mechanical ventilation in COVID‐19 patients.^23^ Our findings support this pattern. PPI use is associated with a greater risk of hospital‐acquired pneumonia as compared with histamine‐H2 receptor antagonists such as famotidine.^24^ As mechanical ventilation and/or vasopressor use is often necessary to treat those with severe pneumonia, this is a reason for the negative associations of greater odds for mechanical ventilation and vasopressor use for pantoprazole.

We found that for acute kidney injury, pantoprazole had higher odds compared to nonuse while famotidine had lower odds compared to pantoprazole. PPIs are positively associated with a subtype of acute kidney injury called acute interstitial nephritis as compared to histamine‐H2 receptor agonists.^25^ Also, acute kidney injury occurs in approximately 20% of hospitalized COVID‐19 patients.^26^ The precise mechanism by which SARS‐CoV‐2 induces acute kidney injury is unknown. Our findings are contrary to those of a previous retrospective study which found that histamine‐H2 receptor antagonists were positively associated with acute kidney injury while no association was found for PPIs.^27^ The possibility of famotidine both reducing viral replication and activating G‐protein‐coupled receptors, thereby preventing pathological vascular inflammation, can be the reason why it was associated with lower odds than pantoprazole for acute kidney injury.

We found that for gastrointestinal bleeding, pantoprazole had higher odds compared to nonuse while famotidine had lower odds compared to pantoprazole. PPIs are typically used for stress ulcer prophylaxis to decrease gastrointestinal bleeding in critically ill patients such as those receiving treatment for septic shock.^28^ Our findings for COVID‐19 are contrary to this pattern. Further verification for this pattern in larger studies is needed.

This study has the strength of comparing two readily available medications from the clinical experience of a healthcare institution. However, study has several limitations. First, as this study was retrospective, we were unable to measure serum biomarkers or viral load in the setting of COVID‐19 infection and consequently could not evaluate famotidine or pantoprazole for anti‐inflammatory or antiviral properties. Second, we did not measure differences in drug dose, route of administration, or timing of therapies in assessing the differences of primary or secondary outcomes. Future research should consider these topics. Third, we did not include data on other possible medications received by patients. Fourth, there is the possibility that selection bias may have confounded the differences in clinical outcomes between famotidine and pantoprazole, as a patient's presentation and need for stress ulcer prophylaxis may have favored the choice of a particular treatment. Future research with a clinical trial would be beneficial to further support the use of famotidine in hospitalized COVID‐19 patients.

## Conclusion

In conclusion, famotidine is associated with lower odds for all‐cause mortality, mechanical ventilation, vasopressor use, acute kidney injury, and gastrointestinal bleeding compared to pantoprazole in patients hospitalized with COVID‐19. The benefits of famotidine are probably due to its immunomodulatory activity during SARS‐CoV‐2 infection, while the benefits of pantoprazole are probably due to its prevention of excessive acid states. The repurposing of medications is frequently more beneficial than developing entirely new medications for any disease because of the immense costs, time constraints, and research processes involved with the latter. We recommend that clinicians consider the use of famotidine over pantoprazole for hospitalized COVID‐19 patients. Future research with a clinical trial would be beneficial to further support the use of famotidine in hospitalized COVID‐19 patients.
